# Bilateral Ovarian Endometriomas Presenting as Nonprogress of Labor: First Case Report in the Literature Is Concomitant Surgical Excision during Cesarean Section Advisable?

**DOI:** 10.1155/2012/741617

**Published:** 2012-11-05

**Authors:** Shashank Shekhar, Chanderdeep Sharma, Vinay Somya, Meghna Thusoo, Nidhi Raina

**Affiliations:** ^1^Department of Obstetrics & Gynecology, Dr. RPGMC Tanda, Kangra 176001, India; ^2^Department of Anesthesia, Dr. RPGMC Tanda, Kangra 176001, India

## Abstract

*Objective*. To report the first case of bilateral ovarian endometriomas, leading to nonprogress of labour, successfully excised during cesarean section. *Design*. Case report. *Setting*. Department of Obstetrics & Gynecology of Dr. RPGMC Tanda, Kangra, India. *Patients*. A primigravida in labour at term gestation. *Interventions*. Surgical management. *Main Outcome Measures*. Description and treatment of a pregnant woman with bilateral ovarian endometriomas during cesarean section. *Results*. Successful excision of ovarian endometriomas and reconstruction of the ovaries during cesarean section. *Conclusion*. Management of incidentally detected endometriomas during cesarean section should be individualized, taking into account the symptoms, size, bilaterality, and adhesion with adjacent organs.

## 1. Introduction

Endometriosis is an estrogen-dependent condition in which ectopic endometrial glands and stroma are found outside the uterus. Most endometrial deposits are found in the pelvis (ovaries, peritoneum, uterosacral ligaments, pouch of Douglas, and rectovaginal septum) [1].The incidence of endometriosis is reported as 4%–17% of all women during their reproductive age. The ovary is variously reported to be involved in 17% to 44% of endometriosis patients [2]. Classical studies suggested that 30% to 50% of women with endometriosis are infertile [3]. Approximately 1% to 4% of pregnant women are diagnosed with an ovarian mass. Of all the adnexal masses reported during pregnancy the incidence of ovarian endometriosis varies widely from 5% to 30% [2, 4]. Surgical noninterference is a widely accepted obstetric policy for ovarian endometriomas during pregnancy; however, literature is quiet regarding the management of incidentally diagnosed endometrioma during cesarean section. We are reporting a case of bilateral ovarian endometriomas leading to nonprogress of labor, which were successfully excised with ovarian reconstruction during cesarean section.

## 2. Case Report

**Figure 1:**
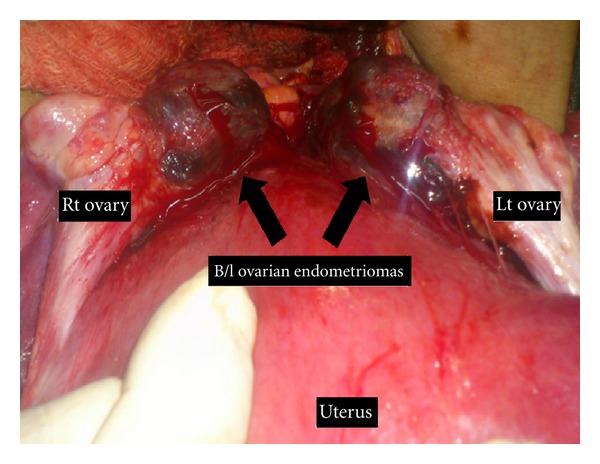
Bilateral ovarian endometriomas (black arrows) adherent to posterior uterine wall partially obstructing the cul-de-sac.

A 32-year-old primigravida at 38 weeks of gestation reported to delivery suite in active labour. She had inadequately supervised antenatal period. No ultrasound scan was done during pregnancy. This was a spontaneous conception after six years of primary infertility. History was also significant for dyspareunia and severe dysmenorrhoea for six years. Her vaginal examination was notable for tender ill-defined mass palpable through posterior fornix. Despite adequate uterine contractions, labour was prolonged and patient was taken up for emergency cesarean section. Lower segment cesarean section was done and a live born healthy male baby weighing 2.7 kg was delivered. Intra-operatively, bilateral ovarian endometriomas measuring 5 × 5 cm each were found stuck in the cul-de-sac. Both the masses were adherent to posterior uterine wall and sigmoid colon, thus partially obliterating the cul-de-sac (Figure 1). According to the American Society for Reproductive Medicine revised classification of endometriosis [5], the patient had stage IV endometriosis. Decision for excision of endometriomas was taken and was successfully performed. Cut section of the cysts revealed chocolate coloured fluid, and histopathological examination confirmed the diagnosis of ovarian endometriosis. Postoperative recovery was unremarkable.

## 3. Discussion

Pregnancy has long been considered as having beneficial effect on the course of endometriosis. As Beecham stated, “Nature (since the beginning of time) has employed an efficient prophylactic and curative measure for endometriosis, that is, pregnancy” [2]. The observed suppressive effect of pregnancy on endometriosis was reviewed by McArthur and Ulfelder in 1965. They showed that pregnancy was frequently accompanied by a reduction in the size of nonovarian endometriotic lesions, although there were notable exceptions [6]. In a recent large retrospective analysis of ovarian endometriosis during pregnancy, it was observed that ovarian endometriotic cysts increased significantly in size in 20% of women and did not change in size in another 28% [2], thus challenging the long held view of beneficial effect of pregnancy on endometriosis. Pregnant women with endometriosis are more likely to suffer from antepartal haemorrhage/placental complications and preeclampsia. Literature suggests that these women are twice more likely to deliver by cesarean section (with no information regarding indications for cesarean section), and the strongest association with endometriosis was observed for prelabour cesarean section [7]. To the best of our knowledge, ovarian endometriomas have previously never been reported to be associated with nonprogress of labor. We hypothesize that large bilateral endometriomas stuck in cul-de-sac prevented descent of head thus leading to nonprogress of labor. Conservative management of ovarian endometriomas during pregnancy is the accepted standard of care [2]. However, management of incidentally found endometriomas during cesarean section is challenging as there is a paucity of literature on the subject matter.We decided to perform concomitant excision of endometriomas during cesarean section in view of long-standing history of severe dyspareunia, and dysmenorrhea as well as it's relatively large size, bilateral nature, and adhesion with surrounding organs.It is difficult to draw any conclusion based upon a single case report; however, we recommend that management of incidentally detected endometriomas during cesarean section should be individualized taking into account the symptoms, size, bilaterality, and adhesion with adjacent organs.
